# Melt-Filled Hard Capsules as an Applicable Compounding Strategy to Enhance the Dissolution of Poorly Water-Soluble Nifedipine Using Polyethylene Glycol Matrices

**DOI:** 10.3390/pharmaceutics18050533

**Published:** 2026-04-27

**Authors:** Nemanja Todorović, Veljko Krstonošić, Milana Vuković, Ivana Zubac, Nataša Milošević, Jelena Jovičić-Bata, Mladena Lalić-Popović

**Affiliations:** 1 Department of Pharmacy, Faculty of Medicine, University of Novi Sad, 21000 Novi Sad, Serbia; nemanja.todorovic@mf.uns.ac.rs (N.T.); veljko.krstonosic@mf.uns.ac.rs (V.K.); ivanazubac154@gmail.com (I.Z.); natasa.milosevic@mf.uns.ac.rs (N.M.); jelena.jovicic-bata@mf.uns.ac.rs (J.J.-B.); mladena.lalic-popovic@mf.uns.ac.rs (M.L.-P.); 2 Centre for Medical and Pharmaceutical Investigations and Quality Control (CEMPhIC), Faculty of Medicine Novi Sad, University of Novi Sad, 21000 Novi Sad, Serbia

**Keywords:** melt-filled capsules, nifedipine, polyethylene glycol, pharmacy compounding, FTIR spectroscopy, dissolution test, rheology, macrogols

## Abstract

**Background/Objectives**: Poor aqueous solubility limits the oral absorption and bioavailability of many active pharmaceutical ingredients. Simple formulation approaches suitable for hospital and community pharmacy compounding are therefore needed. This study aimed to develop and evaluate melt-filled hard capsules containing nifedipine, a model of poorly water-soluble BCS class II drug, using polyethylene glycol (PEG) carriers to improve dissolution performance. **Methods**: PEG blends of different molecular weights (PEG 400, PEG 1500, and PEG 4000) were prepared by melt mixing, followed by incorporation of nifedipine and manual filling into hard gelatin capsules. The formulations were characterized regarding mass variation, drug content, in vitro dissolution, rheological behavior, and solid-state properties using Fourier transform infrared (FTIR) spectroscopy. Dissolution profiles were kinetically modeled and compared with pure nifedipine. **Results**: All capsules met pharmacopoeial requirements for mass uniformity and showed acceptable drug content. PEG-based melt-filled formulations exhibited markedly enhanced dissolution compared with crystalline nifedipine. Faster drug release was associated with lower-molecular-weight PEGs and reduced viscosity, with the PEG 400/PEG 1500 blend demonstrating the most rapid dissolution. Rheological analysis confirmed shear-thinning behavior, while FTIR findings suggested intermolecular interactions and partial amorphization of nifedipine within the PEG matrices. **Conclusions**: This study provides a translational adaptation of solid dispersion principles into a compounding-compatible melt-filling approach.

## 1. Introduction

Poor aqueous solubility remains one of the most significant challenges in the development of oral drug products. Many active pharmaceutical ingredients (APIs) exhibit low water solubility and are classified as Biopharmaceutics Classification System (BCS) class II or IV compounds, where dissolution in gastrointestinal fluids becomes the rate-limiting step for drug absorption and oral bioavailability [1,2]. Consequently, inadequate dissolution often leads to suboptimal therapeutic response and high inter-patient variability, particularly in vulnerable patient populations requiring individualized therapy.

Numerous formulation strategies have been proposed to overcome solubility-limited absorption, including particle size reduction, salt formation, lipid-based systems, molecular complexation, and solid dispersions [3,4,5]. Among these, melt-based approaches are especially attractive due to their solvent-free processing, simplicity, and suitability for both industrial manufacturing and small-scale preparation. Melt processing enables incorporation of poorly soluble drugs into hydrophilic carriers, frequently resulting in reduced crystallinity or amorphization, improved wettability, and enhanced dissolution performance [6]. However, these methods generally rely on specialized extrusion equipment, elevated processing temperatures, and stringent process control, which limit their applicability in decentralized or small-scale pharmaceutical environments [7]. Despite extensive research on melt-based solid dispersions in industrial settings, limited attention has been directed toward simplified melt-filling approaches adaptable to pharmacy compounding practice. Melt-filled hard capsules represent a particularly promising approach in this context. Unlike HME solid dispersion preparation, melt filling can be performed using basic laboratory equipment, making it compatible with pharmacy compounding practice. Furthermore, the growing relevance of melt-based capsule technologies in pharmacy compounding is reflected in recent updates of the German DAC/NRF formulary, an authoritative guideline for magistral preparation in community and hospital pharmacies. The 2023 revision introduced new capsule formulations, updated compounding techniques, and, notably, a dedicated calculation and in-process control guide for melt-filled hard capsules. Such regulatory recognition underscores the practical feasibility and increasing clinical importance of melt-filling approaches within standardized compounding practice [8].

In parallel with technological advances in drug delivery, there is an increasing demand for personalized medicines prepared in hospital and community pharmacies. Pharmacy compounding allows flexible dose adjustment, combination of multiple active substances, and adaptation of dosage forms to the specific needs of pediatric, geriatric, oncological, and veterinary patients, where commercially available products are often unsuitable or unavailable [9,10,11]. Therefore, simple, reproducible, and easily implementable preparation techniques that do not require sophisticated equipment are of particular interest for patient-centered pharmaceutical care. In recent years, there has been growing interest not only in sophisticated industrial technologies but also in simplified melt-based and other dosage forms adaptable to pharmacy practice and personalized medicine. For example, recent work demonstrates the formulation of liquid- and semi-solid filled hard gelatin capsules for patient-tailored therapy, highlighting that such dosage forms can be designed and characterized specifically for compounding environments without bespoke manufacturing infrastructure [12].

Hard capsules filled with molten matrices or semi-solid formulations offer a translatable dosage form that bridges traditional laboratory practice and pharmaceutical compounding by enabling flexible dose adjustment and avoiding complex processing equipment. This aligns with broader trends in personalized medicine, where accessible filling techniques (including use of simple heated fills or semi-automated compounding tools) have been explored as feasible platforms in community and hospital pharmacies. Similarly, comparative investigations of dose-adjustable pantoprazole formulations prepared in compounding settings have emphasized the importance of simple, reproducible preparation techniques that ensure stability while maintaining clinical suitability [13]. These findings underscore the need for solvent-free, accessible, and robust melt-based preparation methods that can be realistically implemented in pharmacy environments.

Hydrophilic carriers such as poly (ethylene glycol) (PEG) 4000 possess favorable physicochemical properties, relatively low melting temperatures, and established safety profiles, supporting their suitability for such applications. Importantly, molecular compatibility between nifedipine and PEG is expected to facilitate partial dispersion or solubilization of the drug within the molten polymer matrix, enhancing the homogeneity of the formulation. Previous investigations have demonstrated that PEG 4000-based solid dispersions of nifedipine can significantly enhance drug solubility, dissolution rate, and systemic exposure, achieving more than a threefold increase in relative bioavailability compared to the pure drug. These findings indicate that the nifedipine–PEG combination represents a rational and mechanistically justified approach for further development within simplified, compounding-adapted melt technologies [14]. Importantly, recent investigations confirm that solid dispersions of poorly soluble drugs with PEG and similar carriers can enhance dissolution and bioavailability in vitro. However, much of this work remains focused on industrially-oriented solid dispersion techniques, while simplified melt-filling into hard capsules has received comparatively limited scientific attention, particularly in the context of pharmacy compounding practice.

Nifedipine, a BCS class II drug widely used in the treatment of cardiovascular disorders, is characterized by low aqueous solubility and dissolution-limited oral absorption [15]. Enhancement of its dissolution rate has been extensively investigated using various solid dispersion systems, including advanced technologies such as supercritical fluid processing, which demonstrated significant improvements in solubility and bioavailability [14]. However, these approaches are primarily designed for industrial manufacturing and are not readily transferable to pharmacy compounding practice. To date, limited attention has been directed toward evaluating simple melt-based filling techniques as a strategy to simultaneously improve nifedipine dissolution and enable flexible, patient-tailored dose preparation. Nifedipine is a lipophilic compound (logP ≈ 2.2–4), which contributes to its poor aqueous solubility. According to the European Pharmacopoeia, nifedipine is classified as practically insoluble in water. Its ionization constants (pKa_1_ ≈ −0.9 and pKa_2_ ≈ 13) lie outside the physiological pH range, resulting in negligible ionization under gastrointestinal conditions. Consequently, nifedipine exhibits pH-independent solubility across the physiological pH range. Its aqueous solubility is extremely low, with reported values of approximately 0.0058 mg/mL at pH 4 and 0.0111 mg/mL at pH 6.8 [14,15]. Nifedipine exhibits a well-defined melting behavior in its pure crystalline powder form, with the most stable polymorph (α_p_) melting at approximately 171 °C, and other polymorphic forms reported to melt between 137 °C and 164 °C. Considering these thermal characteristics, it can be anticipated that, in more complex systems such as molten polyethylene glycol at moderate processing temperatures (60–70 °C), the drug may interact with the carrier, potentially allowing partial dispersion or solubilization. This rationale supports the exploration of melt-based approaches as a means to enhance dissolution while avoiding high-temperature processing [16].

Accordingly, the aim of the present study was to develop and characterize melt-filled hard capsules containing nifedipine and PEG mixtures as a hydrophilic carrier, prepared using a melt-based filling technique suitable for pharmacy compounding. The formulations were evaluated in terms of physicochemical and solid-state properties, dissolution, and rheological behavior in order to assess their potential as an applicable and effective strategy for patient-tailored pharmacy preparation.

## 2. Materials and Methods

### 2.1. Materials

Micronized nifedipine complying with the requirements of the European Pharmacopoeia, 11th edition (Ph. Eur. 11), was obtained from Farmalabor (Italy). Polyethylene glycol 400 (PEG 400), polyethylene glycol 1500 (PEG 1500), and polyethylene glycol 4000 (PEG 4000) were purchased from Alfa Aesar (Kandel, Germany). Hard gelatin capsules (size 0; Farmalabor, Canosa di Puglia, Italy) contained indigo carmine (FD&C Blue 2, E132), titanium dioxide, yellow iron oxide (E172), and gelatin. Methanol (Lachner, Neratovice, Czech Republic) was used for content determination. Sodium lauryl sulfate (SLS; Centrohem, Stara Pazova, Serbia) was used as the dissolution medium in the form of a 0.5% (*w*/*v*) aqueous solution. Purified water obtained by distillation (AC-L8, Optic Ivymen System, J.P. Selecta, Barcelona, Spain) was used throughout all experimental procedures. The selection of PEG types (PEG 400, 1500, and 4000) and their ratios was designed to enhance the dissolution rate of nifedipine. PEG 400 was included in higher proportions due to its lower molecular weight and higher solubility, which were expected to accelerate drug release. The content of low molecular weight PEG (PEG 400) was limited to ≤50% to ensure gelatin capsule stability, as higher concentrations have been reported to compromise capsule integrity [17].

### 2.2. Preparation of Melt-Filled Capsules

PEG blends were prepared by melt mixing at the lowest possible temperature. Melting was performed in glass beakers placed in a thermostatically controlled shaking water bath (WSB-18, Witeg, Wertheim, Germany) at 60 °C for the PEG 1500 series and 70 °C for the PEG 4000 series.

The molten mixtures were transferred to a graduated cylinder of appropriate volume, and previously weighed nifedipine powder was added. Stirring was continued until complete dissolution of the drug, as indicated by clarification of the melt. After dissolution, the mixtures were adjusted to the required final volume with the corresponding molten PEG blend. Capsules were filled by transferring a precise volume of the molten PEG–nifedipine mixture using a pipette, which inherently minimized operator-dependent variability. Although this method is time-consuming, it is suitable for small-scale, compounding-oriented studies. Future work may implement semi- or fully automated filling systems to increase throughput and further improve reproducibility

Approximately 70% of the capsule volume was targeted for filling, corresponding to 475 µL of the prepared melt per capsule. Capsules were filled manually using a capsule-filling device (Farmalabor, Canosa di Puglia, Italy) and by transferring a precise volume of the molten PEG–nifedipine mixture using a pipette, which inherently minimized operator-dependent variability. Although this method is time-consuming, it is suitable for small-scale, compounding-oriented studies. Future work may implement semi- or fully automated filling systems to increase throughput and further improve reproducibility.

The compositions of the prepared formulations (expressed per capsule) are presented in Table 1. As the objective of the study was not to introduce a new dose, but to develop an alternative formulation based on melt-filled capsules, we selected one dose of nifedipine (20 mg), which corresponds to a clinically established strength available in conventional dosage forms. Due to the known photosensitivity of nifedipine, all preparation and testing procedures were performed under light-protected conditions to minimize exposure to natural and artificial light. Photographs of the prepared capsules are provided in the Supplementary Material (Figure S1).

### 2.3. Content Determination

The nifedipine content was determined in ten randomly selected capsules. The capsule contents were dissolved in methanol and sonicated for 30 min at room temperature using an ultrasonic bath (Bandelin, Germany) to ensure complete extraction of the drug.

Quantification was performed spectrophotometrically (Agilent 8453, Santa Clara, CA, USA) by measuring the absorbance at the wavelength of maximum absorption [18,19]. Linearity of the method was confirmed over the concentration range of 0.75125–50 µg/mL (R^2^ = 0.9998).

### 2.4. Mass Variation

The prepared capsules were evaluated in accordance with the Ph. Eur. 11 test for uniformity of mass of single-dose preparations.

The mass of twenty capsules was determined using an analytical balance (Radwag, Radom, Poland) before and after careful removal of the capsule contents. The net fill mass was calculated for each unit, and the mean mass was determined. Individual masses were compared with the calculated mean value.

The permitted deviation was ±7.5% for dosage forms with an average mass greater than 300 mg.

### 2.5. Dissolution Testing

In vitro dissolution testing was performed using a dissolution apparatus (Erweka DT800, Heussenstam, Germany) equipped with Apparatus I (basket method). The temperature was maintained at 37 ± 0.5 °C with a rotation speed of 100 rpm. A 0.5% (*w*/*v*) aqueous SLS solution was used as the dissolution medium.

Samples (5 mL) were withdrawn at predetermined time points (5, 15, 25, 35, 45, and 60 min) and immediately replaced with an equal volume of fresh medium preheated to 37 °C. Dissolution testing was performed using six capsules per formulation (*n* = 6). Nifedipine concentrations were determined spectrophotometrically.

Dissolution data were modeled and compared using DDSolver add-in software (version 2007) [20]. Time (min) and cumulative percentage of drug released were used as input parameters. The goodness of fit of several kinetic models (logistic and Weibull models) was evaluated based on visual inspection and statistical criteria, including the adjusted coefficient of determination (R^2^_adj), Akaike information criterion (AIC), and model selection criterion (MSC) [20].

Similarity between the dissolution profiles of PEG-based formulations (M1–M6) and the reference formulation containing pure nifedipine (M0) was assessed using the similarity factor (f2). Dissolution profiles were considered similar when f2 values were greater than 50. Samples were stored in tightly closed, light-protected containers at room temperature, and dissolution testing was repeated after one month to evaluate stability. Additionally, the solubility of nifedipine was determined by the shake-flask method in 0.5% SLS at 37 °C. An excess amount of nifedipine was added, and the suspension was shaken for 24 h in a shaking water bath (WSB-18, Witeg, Wertheim, Germany). The supernatant was then filtered and analyzed spectrophotometrically. Subsequently, the same samples were kept under static conditions at room temperature for an additional 24 h in order to evaluate their stability.

### 2.6. Rheological Characterization

Rheological measurements of the prepared formulations were performed using a HAAKE MARS rheometer (Thermo Scientific, Karlsruhe, Germany). A parallel-plate geometry (P35 Ti, 1.0 mm gap) was used at the respective filling temperatures: 60 °C for the PEG 1500 series (M1–M3) and 70 °C for the PEG 4000 series (M4–M6).

Flow curves were obtained by gradually increasing the shear rate from 0.001 to 200 s^−1^ over 120 s, followed by a constant shear phase of 60 s and a subsequent decrease to 0 s^−1^ over 120 s. Oscillatory measurements did not reveal a linear viscoelastic region for any formulation.

Data analysis was performed using HAAKE RheoWin 4 Data Manager software (4.91.0011). The experimental flow behavior was best described by the power-law model:

$$\tau =K{\left(\dot{\gamma }\right)}^{n}$$

where τ is the shear stress (Pa), K is the consistency coefficient in (Pa s^n^), $\dot{\gamma }$ is the shear rate (s^−1^), n is the flow-behavior index (dimensionless).

Model parameters were compared using one-way analysis of variance (ANOVA) followed by Tukey’s post hoc test in SPSS (IBM SPSS Statistics v26, Chicago, IL, USA). Differences were considered statistically significant at *p* < 0.05.

### 2.7. Fourier Transform Infrared (FTIR) Spectroscopy

FTIR spectra of the raw materials and prepared formulations were recorded using an FTIR spectrophotometer (Nicolet iS10, Thermo Scientific, Waltham, MA, USA). Data acquisition and processing were performed using OMNIC 8.1 software (Thermo Scientific, Waltham, MA, USA).

Background spectra were collected in air prior to each measurement. Samples were scanned 32 times over the spectral range of 4000–400 cm^−1^ with a resolution of 4 cm^−1^. Spectra were normalized to the most intense peak to allow direct visual comparison across samples.

## 3. Results

### 3.1. Mass Variation and Content Determination

The results of the mass variation of the prepared formulations and nifedipine content uniformity (individual content vs. average content of the sample) are presented in Table 2. All prepared capsules complied with the Ph. Eur. 11 requirements for the uniformity of mass test, as the mass of each individual unit remained within the permitted deviation range (±7.5% of the mean mass).

The average nifedipine content ranged from 95.08% to 104.13% of the labeled amount, confirming satisfactory content uniformity and accurate dosing of the melt-filled capsules.

### 3.2. Dissolution Testing

The solubility of nifedipine in 0.5% SLS at 37 °C was 224.6 ± 32.7 µg/mL (*n* = 6). After 24 h under the same conditions, the concentration increased to 243.59 ± 5.96 µg/mL (*n* = 6). Although a higher concentration was observed after 24 h, the difference was not statistically significant (*t*-test, *p* = 0.3776), indicating stability of nifedipine in the tested medium. The determined solubility of nifedipine in 0.5% SLS at 37 °C indicates the presence of sink conditions when testing a 20 mg dose in a volume of 900 mL.

**Figure 1:**
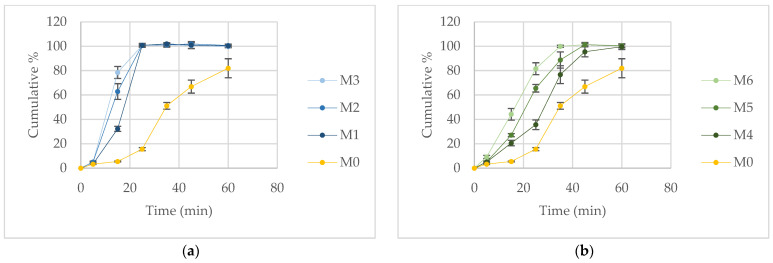
In vitro dissolution profiles of nifedipine from the prepared melt-filled capsule formulations compared with pure nifedipine (M0): (**a**) PEG 1500-based formulations (M1–M3); (**b**) PEG 4000-based formulations (M4–M6). Data are presented as mean ± SD (*n* = 6).

The dissolution profiles presented in Figure 1 demonstrate a pronounced improvement in the dissolution rate of nifedipine from the prepared formulations compared with the pure drug.

After 15 min, formulation M3 released 78.47 ± 4.93% of nifedipine, followed by M2 (62.86 ± 6.46%), M6 (44.24 ± 4.76%), M1 (32.23 ± 2.08%), M5 (27.09 ± 1.34%), and M4 (20.56 ± 2.24%).

Within 25 min, formulations M1, M2, and M3 achieved complete drug release (≈100%), while M6 released 81.58 ± 4.89%, M5 65.55 ± 3.11%, and M4 35.65 ± 3.95%. Complete release from M6 and M5 was reached after 35 and 45 min, respectively.

In contrast, the reference formulation containing pure nifedipine (M0) exhibited markedly slower dissolution, releasing only 5.44 ± 0.42% after 15 min, 15.60 ± 1.28% after 25 min, and 81.97 ± 7.77% after 60 min.

Kinetic modeling of the dissolution data indicated that the release profiles of M0 and M4 were best described by the logistic-3 model. Formulation M1 followed the logistic-2 model, whereas formulations M2 and M3 were best fitted by the logistic-1 model. Although the dissolution profiles of M1–M3 differ only slightly at one time point, the model parameters vary among these formulations, as summarized in Table 3, supporting the selection of distinct kinetic models for each formulation. The dissolution behavior of formulations M5 and M6 was most adequately described by the Weibull model. The corresponding model parameters are summarized in Table 3.

The calculated similarity (f2) factors for comparison of the PEG-based formulations with the reference formulation containing pure nifedipine (M0) are presented in Table 4.

All tested formulations exhibited f2 values outside the generally accepted similarity range, indicating dissolution behavior distinct from that of pure nifedipine. These results further confirm the substantial modification of the drug release performance achieved by incorporation of PEG-based melt matrices. Dissolution profiles measured immediately after preparation and after one month of storage showed no statistically significant differences (Supplementary Material, Figure S2). The similarity factors (f_2_) for the six formulations were 60.21, 50.86, 52.05, 54.14, 52.43, and 52.86, confirming comparable dissolution behavior over the storage period.

### 3.3. Rheological Properties

**Figure 2:**
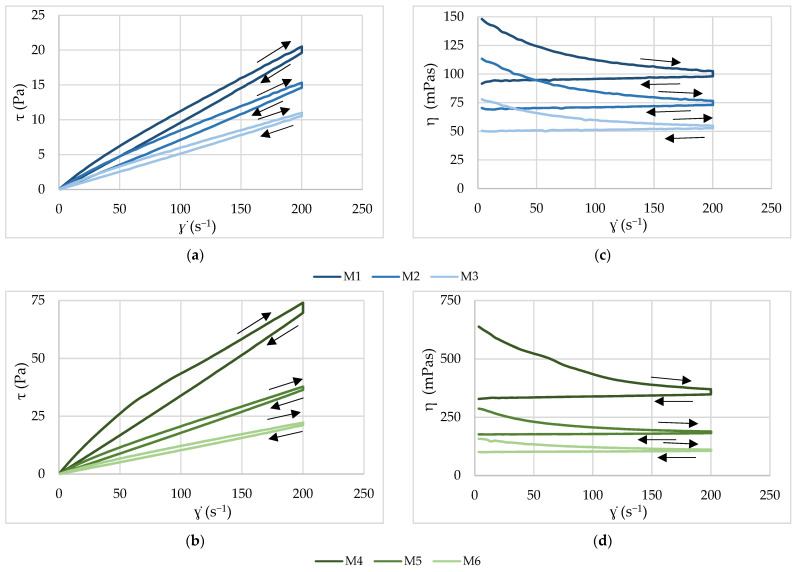
Flow curves (shear stress vs. shear rate) of the prepared formulations: (**a**) PEG 1500 series (M1–M3); (**b**) PEG 4000 series (M4–M6), and apparent viscosity as a function of shear rate: (**c**) PEG 1500 series (M1–M3); (**d**) PEG 4000 series (M4–M6).

The flow curves of the prepared formulations are shown in Figure 2. It is obvious that all formulations showed non-Newtonian flow behavior. They exhibited time-dependent thixotropic flow behavior, with pronounced shear-thinning characteristics for upward curves (Table 5). The general shape of the curves did not change markedly with variations in the proportion of individual PEG components. Thixotropic flow behavior was particularly pronounced for formulation M4 containing 100% PEG 4000.

A decreasing trend in shear stress across all shear rates was observed with increasing PEG 400 content in the formulations.

The lowest shear stress values as well as apparent viscosities were recorded for formulation M3, whose flow profile was the closest to Newtonian behavior. The parameters of the mathematical model providing the best fit to the experimental flow curves are summarized in Table 5. Consistency coefficient (K) is an indicator of the viscosity of the formulations, and the flow behavior index (n) describes the extent of non-Newtonian behavior of the formulations. The values of index n confirm the shear-thinning character of the formulations, while the values of the hysteresis loop areas show that the highest one has the formulation that shows the highest consistency coefficient and apparent viscosity from Figure 2 (M4). Also, it is obvious that the hysteresis loop area decreases as the apparent viscosity of the formulations decreases.

### 3.4. Fourier Transform Infrared (FTIR) Spectroscopy

**Figure 3:**
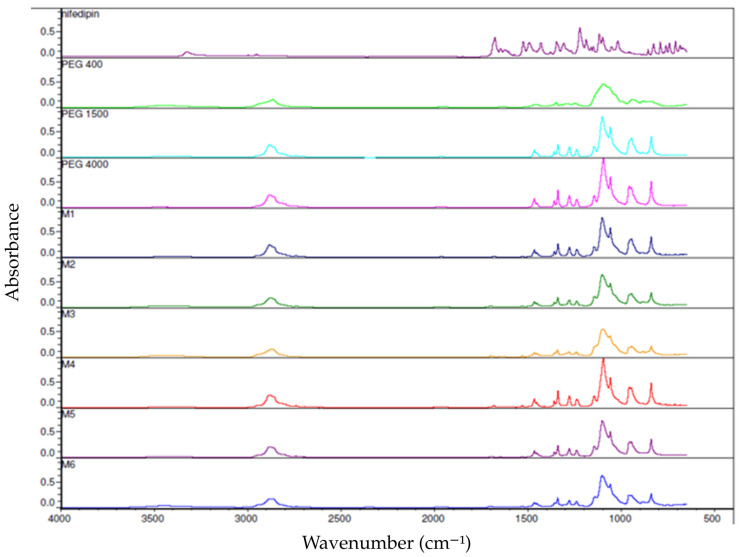
FTIR spectra of pure nifedipine, PEG 400, PEG 1500, PEG 4000, and the corresponding melt-filled capsule formulations.

The FTIR spectra of pure nifedipine, PEG 400, PEG 1500, PEG 4000, and the prepared formulations are presented in Figure 3.

The spectrum of pure nifedipine exhibited characteristic absorption bands at approximately 1225 cm^−1^ (C–O stretching), 1350 cm^−1^ (NO_2_ stretching), 1650 cm^−1^ (C=C stretching), 1680 cm^−1^ (ester C=O stretching), and 3300 cm^−1^ (N–H stretching).

The spectra of PEGs showed a strong and sharp band around 1100 cm^−1^ corresponding to ether (C–O–C) stretching vibrations, as well as bands near 2900 cm^−1^ attributed to aliphatic C–H stretching.

In the spectra of the nifedipine–PEG formulations, a slight shift and broadening of the band at approximately 3300 cm^−1^, together with a decrease in intensity of the band at 1680 cm^−1^, were observed. No new absorption bands appeared in the spectra of the formulations.

## 4. Discussion

This study demonstrated that melt-filled hard capsules represent an applicable and effective compounding strategy for improving the dissolution performance of poorly water-soluble API, nifedipine. Incorporation of nifedipine into PEG-based molten matrices resulted in markedly faster and more complete drug release compared with the pure drug, while maintaining acceptable mass and content uniformity. In addition to enhanced biopharmaceutical performance, the melt-filling approach enabled straightforward preparation using minimal equipment, supporting its suitability for small-scale and pharmacy-based production. These findings highlight the potential of melt-filled capsules as a practical approach for personalized oral dosage forms, particularly for drugs limited by dissolution-controlled absorption.

The prepared melt-filled capsules demonstrated satisfactory pharmaceutical quality in terms of both mass uniformity and drug content. All units complied with the requirements of the Ph. Eur. 11. Chapter 2.9.5. [21], as individual capsule weights remained within ±7.5% of the mean mass. This confirms the reproducibility of the manual filling procedure despite the molten nature of the formulation.

The nifedipine content (95.08–104.13% of the labeled amount) was within commonly accepted regulatory limits for finished pharmaceutical products. In accordance with Directive 2001/83/EC, assay specifications for medicinal products must be defined and justified in the product dossier; in practice, a ±5% range around the label claim is widely accepted for immediate-release solid dosage forms [22]. The obtained results therefore indicate adequate dosing accuracy and homogeneous drug distribution within the PEG-based matrices.

The dissolution test clearly demonstrated that both the type and the proportion of the polymeric carrier significantly affected the release behavior of nifedipine from the prepared formulations (Figure 1). The dissolution rate of pure nifedipine in the selected medium was low, which is consistent with previously published findings [15,23]. It has been established that the formation of polymer-based solid or molecularly dispersed systems has been reported to enhance the dissolution rate of the drug compared with crystalline nifedipine. Water-soluble polymers such as PEGs can markedly improve drug solubility and dissolution, particularly when intermolecular interactions with the API occur. The effectiveness of enhancing the dissolution rate of nifedipine through solid dispersion technology has been previously demonstrated using various preparation methods and different polymeric carriers [14,19,24]. It was observed that increasing the proportion of lower-molecular-weight PEGs resulted in a faster dissolution of nifedipine. Polymers with lower molecular weight, such as PEG 400 and PEG 1500, exhibited improved wettability and lower viscosity, facilitating more rapid penetration of the dissolution medium and enhanced contact between the solvent and drug particles, ultimately leading to accelerated drug release. Formulation M3 (containing PEG 400 and PEG 1500 in a 1:1 ratio) exhibited the highest dissolution rate (Figure 1) and simultaneously showed the lowest viscosity among the tested systems (Figure 2).

The influence of viscosity on dissolution behavior can be attributed to the fact that, in highly viscous dispersions, drug particles may become entrapped within the viscous matrix, which hinders drug release and reduces the dissolution rate. In contrast, lower-viscosity systems allow faster penetration of the dissolution medium and improved dispersion of drug particles, thereby facilitating more rapid drug release [25]. Accordingly, increasing the proportion of higher-molecular-weight PEGs resulted in increased viscosity and a corresponding decrease in nifedipine dissolution. All investigated formulations exhibited a decrease in apparent viscosity with increasing shear rate (shear-thinning behavior), along with time-dependent thixotropic behavior. Such behavior is characteristic of polymer melts and reflects alignment and partial disentanglement of macromolecular chains under shear stress, leading to viscosity reduction [19]. Shear-thinning is technologically advantageous for melt-filled capsule preparation, as it enables easier dosing under applied shear while maintaining higher apparent viscosity at rest, thereby supporting formulation stability during handling. The consistency index (K) increased with higher PEG molecular weight, with formulation M4 (100% PEG 4000) demonstrating markedly higher viscosity compared with PEG 1500- and PEG 400-containing systems. This trend is consistent with established polymer physics principles, where increasing molecular weight enhances chain entanglement density and resistance to flow [19]. Similar behavior has been described for melt-based solid dispersion systems used to enhance the performance of poorly soluble drugs [14]. Furthermore, the formulations also showed a time-dependent, thixotropic behavior with a pronounced hysteresis loop. The reason for the thixotropic behavior is the three-dimensional structure that the system has at rest, which in this case originates from the PEG polymer chains. When a stress is applied on such a system, the three-dimensional structure is disturbed, which leads to a decrease in viscosity (shear-thinning behavior). Partial recovery of the structure after the reduction of the stress effect (downward curve) leads to the appearance of a hysteresis loop. The hysteresis loop area shows the energy required to break down the structure of the system [26]. The larger hysteresis loop area observed for PEG 4000-rich systems suggests greater structural resistance under shear, potentially reflecting higher entanglement density within the melt. These findings indicate that optimization of PEG molecular weight and blend composition is critical not only for dissolution enhancement but also for ensuring appropriate processability in compounding-adapted melt systems.

The FTIR spectra of the starting materials (nifedipine, PEG 400, PEG 1500, and PEG 4000) (Figure 3) were consistent with previously reported data in the literature [27]. The spectra of the melt-filled nifedipine–PEG formulations largely retained the characteristic bands of the individual components. No new absorption peaks were observed, indicating the absence of chemical degradation or formation of new covalent species during the melt-filling process. However, a decrease in the intensity of the band at approximately 1680 cm^−1^ and a shift and broadening of the band around 3300 cm^−1^ were detected in the formulations. These changes suggest intermolecular interactions between nifedipine and PEG, most likely hydrogen bonding. Such interactions may contribute to partial amorphization or molecular dispersion of nifedipine within the PEG matrix and to stabilization of the non-crystalline state. Our results are in accordance with previous studies where shown that FTIR spectra of PEG samples with different molecular weights (e.g., PEG400 and PEG4000) display the characteristic ether and hydroxyl bands, with minor shifts in peak positions likely due to differences in chain length and number of repeating units [17].

Amorphous or molecularly dispersed forms of a drug possess higher free energy compared with their crystalline counterparts due to the absence of a long-range ordered lattice. Consequently, less energy is required for the transition from the solid to the dissolved state, which facilitates faster dissolution. In addition, weaker intermolecular interactions in the non-crystalline state allow more rapid solvent penetration into the matrix, further contributing to enhanced dissolution performance [28,29].

## 5. Conclusions

The results of the performed investigations demonstrate that the use of PEG blends with different molecular weights as melt-based matrices significantly influences the dissolution behavior of nifedipine. The dissolution rate of nifedipine was markedly enhanced in all tested PEG-containing formulations compared with the pure crystalline drug, with both the type and molecular weight of PEG playing a decisive role.

Lower-molecular-weight PEG blends provided reduced melt viscosity, improved processability, and accelerated drug release, while higher-molecular-weight systems exhibited increased structural resistance and slower dissolution. The combined dissolution, rheological, and FTIR findings suggest that improved performance results from enhanced drug dispersion within the hydrophilic matrix and partial reduction of crystallinity.

Importantly, the proposed melt-filling approach requires minimal equipment and is compatible with pharmacy compounding practice. This positions melt-filled PEG-based capsules as a practical translational approach bridging solid dispersion principles with individualized, small-scale pharmaceutical preparation.

## Figures and Tables

**Table 1:** Characteristics and composition of the investigated formulations.

Formulation	Composition of the Matrix (%)			Nifedipine (mg per Capsule)	Preparation Temperature (°C)
	PEG 400	PEG 1500	PEG 4000		
M0	–	–	–	20	25
M1	–	100	–	20	60
M2	25	75	–	20	60
M3	50	50	–	20	60
M4	–	–	100	20	70
M5	25	–	75	20	70
M6	50	–	50	20	70

**Table 2:** Mass variation and nifedipine content of the prepared melt-filled capsules.

Formulation	Average Mass (mg)	Mass Variation (% of Average Mass)		Nifedipine Content (% of Label Claim, Average ± SD)
		Min	Max	
M1	545.23	94.09	106.74	98.12 ± 3.04
M2	548.22	93.27	105.23	99.84 ± 2.17
M3	554.29	94.59	102.42	97.69 ± 2.50
M4	536.41	92.76	107.38	99.03 ± 3.75
M5	530.20	93.32	106.83	100.47 ± 3.66
M6	547.73	93.86	102.79	99.25 ± 2.48

**Table 3:** Parameters of the kinetic models providing the best fit to the experimental dissolution data.

Formulation	Kinetic Models							
	Logistic Models 1 and 2			Logistic 3			Weibull 2	
	α	β	Fmax	Fmax	k	γ	α	β
M0				0 ± 0	35 ± 1	86 ± 8		
M1	−8 ± 1	7 ± 0	109 ± 2					
M2	−9 ± 1	8 ± 1	–					
M3	−9 ± 0	9 ± 1	–					
M4				0 ± 0	29 ± 1	105 ± 2		
M5							547 ± 47	2 ± 0
M6							224 ± 93	2 ± 0

**Table 4:** Similarity (f2) factors for comparison of dissolution profiles with the reference formulation (M0).

Formulation	f2
M1	19
M2	17
M3	16
M4	37
M5	27
M6	21

**Table 5:** Rheological parameters of the power-law model fitted to the experimental flow curves (mean ± SD).

Formulation	Increasing Shear Rate		Decreasing Shear Rate		Hysteresis Loop Area (P·s^−1^)
	K	*n*	K	*n*	
M1	0.21270 ± 0.00269	0.85977 ± 0.00437	0.08348 ± 0.00213 ^b,c^	1.02733 ± 0.00321	270.07 ± 8.16
M2	0.15790 ± 0.00877	0.86247 ± 0.01315	0.06174 ± 0.00261 ^a,c^	1.03067 ± 0.00153	202.17 ± 17.93
M3	0.10860 ± 0.00145	0.86997 ± 0.00111	0.04555 ± 0.00065 ^a,b^	1.02800 ± 0.00100	134.27 ± 0.75
M4	1.12110 ± 0.17431 ^e,f^	0.79190 ± 0.02837 ^e,f^	0.30447 ± 0.00517 ^e,f^	1.02733 ± 0.00306	1283.00 ± 230.63 ^e,f^
M5	0.41803 ± 0.01790 ^d^	0.85087 ± 0.00090 ^d^	0.16320 ± 0.00183 ^d,f^	1.02267 ± 0.00252	502.53 ± 42.28 ^d^
M6	0.22907 ± 0.02132 ^d^	0.87203 ± 0.01751 ^d^	0.09583 ± 0.00457 ^d,e^	1.02633 ± 0.00115	301.80 ± 38.84 ^d^

^a–f^ Values are expressed as mean ± SD (*n* = 3). Different superscript letters indicate statistically significant differences (one-way ANOVA with Tukey’s post hoc test, *p* < 0.05): ^a^ vs. M1; ^b^ vs. M2; ^c^ vs. M3; ^d^ vs. M4; ^e^ vs. M5; ^f^ vs. M6.

## Data Availability

The original contributions presented in the study are included in the article, further inquiries can be directed to the corresponding author.

## Supplementary Materials

The following supporting information can be downloaded at: https://www.mdpi.com/article/10.3390/pharmaceutics18050533/s1, Figure S1. Photographs of the prepared capsules and fragments of the cooled filling (one month after preparation). Figure S2. Dissolution profiles of prepared capsules at preparation (orange) and after one month storage (blue) under controlled conditions. Panels a-f correspond to formulations M1–M6

## Abbreviations

The following abbreviations are used in this manuscript:

AIC	Akaike information criterion
API	Active pharmaceutical ingredient
BCS	Biopharmaceutics Classification System
FTIR	Fourier transform infrared
HME	Hot-melt extrusion
MSC	Model selection criterion
Ph. Eur. 11	European Pharmacopoeia 11th Edition
PEG	Polyethylene glycol
SLS	Sodium lauryl sulfate
