# Supplementary material for: Melt-Filled Hard Capsules as an Applicable Compounding Strategy to Enhance the Dissolution of Poorly Water-Soluble Nifedipine Using Polyethylene Glycol Matrices
Source: Pharmaceutics. 2026 Apr 27;18(5):533. doi: 10.3390/pharmaceutics18050533 (PMC13210443; doi:10.3390/pharmaceutics18050533)
Supplement: Supplementary file 1 [file pharmaceutics-18-00533-s001.zip › pharmaceutics-4215470-supplementary.pdf]

Supplementary Material

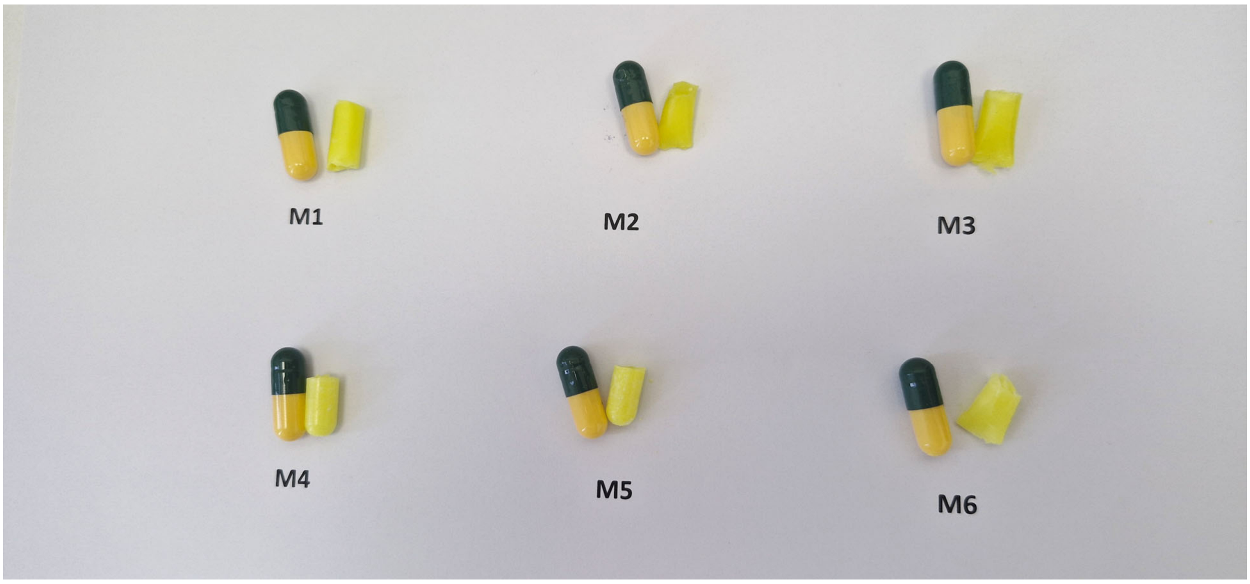

Figure S1. Photographs of the prepared capsules and fragments of the cooled filling (one month after preparation).

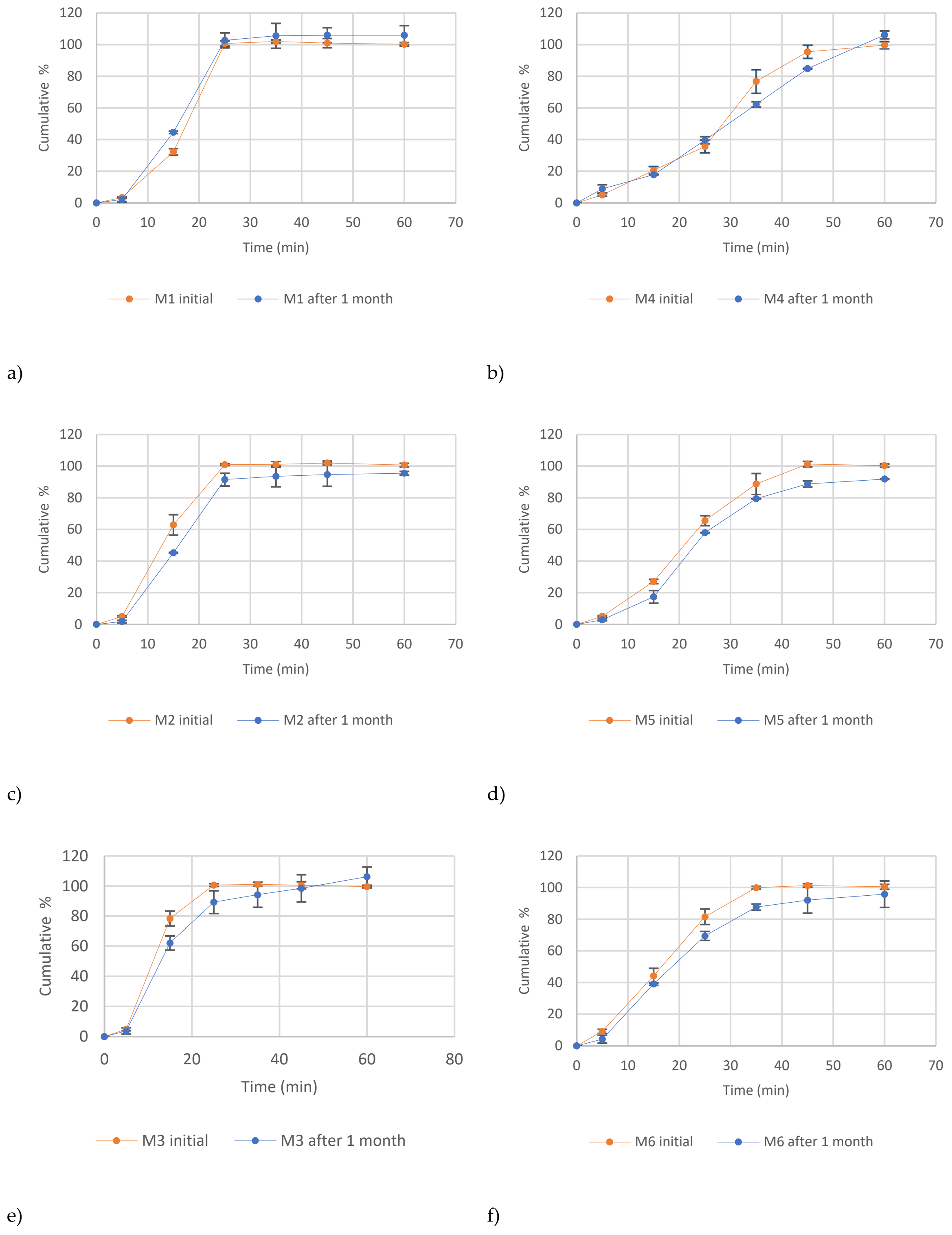

Figure S2. Dissolution profiles of prepared capsules at preparation (orange) and after one month storage (blue) under controlled conditions. Panels a-f correspond to formulations M1–M6.
